# Neuropsychiatric symptoms are early indicators of an upcoming metabolic decline in Alzheimer’s disease

**DOI:** 10.1186/s40035-020-00225-y

**Published:** 2021-01-04

**Authors:** Kok Pin Ng, Tharick A. Pascoal, Sulantha Mathotaarachchi, Yiong Huak Chan, Lai Jiang, Joseph Therriault, Andrea L. Benedet, Monica Shin, Nagaendran Kandiah, Celia M. T. Greenwood, Pedro Rosa-Neto, Serge Gauthier, Dominantly Inherited Alzheimer Network

**Affiliations:** 1 Alzheimer’s Disease Research Unit, McGill Centre for Studies in Aging, McGill University, Montréal, Québec Canada; 2 Translational Neuroimaging Laboratory, The McGill University Research Centre for Studies in Aging, Montreal, Canada; 3 Department of Neurology, National Neuroscience Institute, Singapore City, Singapore; 4 Biostatistics Unit, Yong Loo Lin School of Medicine, National University of Singapore, Singapore City, Singapore; 5 Lady Davis Institute, McGill University, Montreal, Canada; 6 Department of Epidemiology, Biostatistics and Occupational Health, McGill University, Montreal, Canada

**Keywords:** Neuropsychiatric symptoms, Dominantly inherited Alzheimer’s disease, Metabolic correlates

## Abstract

**Background:**

Neuropsychiatric symptoms (NPS) are increasingly recognized as early non-cognitive manifestations in the Alzheimer’s disease (AD) continuum. However, the role of NPS as an early marker of pathophysiological progression in AD remains unclear. Dominantly inherited AD (DIAD) mutation carriers are young individuals who are destined to develop AD in future due to the full penetrance of the genetic mutation. Hence, the study of DIAD mutation carriers enables the evaluation of the associations between pure AD pathophysiology and metabolic correlates of NPS without the confounding effects of co-existing pathologies. In this longitudinal study, we aimed to identify regional brain metabolic dysfunctions associated with NPS in cognitively intact DIAD mutation carriers.

**Methods:**

We stratified 221 cognitively intact participants from the Dominantly Inherited Alzheimer’s Network according to their mutation carrier status. The interactions of NPS measured by the Neuropsychiatric Inventory-Questionnaire (NPI-Q), age, and estimated years to symptom onset (EYO) as a function of metabolism measured by [^18^F]flurodeoxyglucose ([^18^F]FDG) positron emission tomography, were evaluated by the mixed-effects regression model with family-level random effects in DIAD mutation carriers and non-carriers. Exploratory factor analysis was performed to identify the neuropsychiatric subsyndromes in DIAD mutation carriers using the NPI-Q sub-components. Then the effects of interactions between specific neuropsychiatric subsyndromes and EYO on metabolism were evaluated with the mixed-effects regression model.

**Results:**

A total of 119 mutation carriers and 102 non-carriers were studied. The interaction of higher NPI-Q and shorter EYO was associated with more rapid declines of global and regional [^18^F]FDG uptake in the posterior cingulate and ventromedial prefrontal cortices, the bilateral parietal lobes and the right insula in DIAD mutation carriers. The neuropsychiatric subsyndromes of agitation, disinhibition, irritability and depression interacted with the EYO to drive the [^18^F]FDG uptake decline in the DIAD mutation carriers. The interaction of NPI and EYO was not associated with [^18^F]FDG uptake in DIAD mutation non-carriers.

**Conclusions:**

The NPS in cognitively intact DIAD mutation carriers may be a clinical indicator of subsequent metabolic decline in brain networks vulnerable to AD, which supports the emerging conceptual framework that NPS represent early manifestations of neuronal injury in AD. Further studies using different methodological approaches to identify NPS in preclinical AD are needed to validate our findings.

## Background

Neuropsychiatric symptoms (NPS) are frequently observed in mild cognitive impairment (MCI) and dementia stages of Alzheimer’s disease (AD) [1–4] and are associated with greater functional impairment, poorer quality of life, accelerated cognitive decline [5–7] and a more significant degree of AD neurodegeneration [4]. In cognitively normal individuals, developing NPS later in life may potentially increase the risk of cognitive decline [8–11]. Therefore, NPS are being increasingly considered as a non-cognitive manifestation in the early stages of AD when one is cognitively intact [12]. However, the roles of NPS as early clinical manifestations of pathophysiological progression of AD in cognitively normal individuals remain unclear. Our recent study showed that NPS in preclinical sporadic AD individuals preceded hypometabolism in the posterior cingulate cortex, a key brain region involved in the AD process [13]. Hence, investigating NPS as an early manifestation of metabolic decline in an independent cognitively intact cohort known to have AD pathophysiology will further advance the emerging conceptual framework in which NPS constitute an early clinical manifestation of AD.

Dominantly inherited AD (DIAD) is a familial AD due to autosomal dominant mutations in *APP*, *PSEN1* or *PSEN2* and cognitively intact individuals who are DIAD mutation carriers are destined to develop AD in future due to the full penetrance of the genetic mutation [14]. Similar to the late-onset sporadic AD [15], the pathophysiology of DIAD begins to accumulate in the preclinical stage of the disease when the carriers are cognitively normal [16, 17]. In DIAD, early behavioural changes have been reported in mutation carriers with mild cognitive symptoms, in whom the NPS increase as their disease progresses [18]. Furthermore, the DIAD mutation carriers are younger than individuals with sporadic AD and are less likely to have other medical conditions such as cerebrovascular diseases [19]. Therefore, by enrolling cognitively normal DIAD mutation carriers, the associations between pure AD pathophysiology and metabolic correlates of NPS in the preclinical stage of AD can be evaluated without the confounding effects of co-existing pathologies.

In this study, we set out to perform this observation in cognitively normal DIAD mutation carriers and non-carriers from the Dominantly Inherited Alzheimer Network (DIAN) [20].

## Methods

### Participants

Data analyzed in this study were obtained from the DIAN Data Freeze 11. The DIAN observational study is an international multi-site study that enrolls family members who have parents with a mutated gene known to cause DIAD [20]. The study participants may or may not be mutation carriers and they may or may not have cognitive symptoms. The participants underwent standardized clinical and cognitive testing, brain imaging, and biological fluid collection (blood, cerebrospinal fluid [CSF]) to determine the sequence of changes in pre-symptomatic mutation carriers who are destined to develop AD.

In this study, we selected cognitively normal DIAD mutation carriers and non-carriers from the DIAN cohort with clinical dementia rating (CDR) [21] score of 0 and mini-mental state examination [22] score ≥ 24 [18, 23]. Individuals who were mildly symptomatic (CDR = 0.5) or overtly symptomatic (CDR > 0.5) were excluded. The results of neuropsychiatric inventory-questionnaire (NPI-Q) and [^18^F]Flurodeoxyglucose (FDG) positron emission tomography (PET) performed at the first visit and at subsequent yearly follow-ups (if available) for each participant were analyzed.

### Ethical approval and patient consent

The DIAN study was approved by the Institutional Review Boards of all of the participating institutions. Informed written consent was obtained from all participants at each site.

### Neuropsychiatric assessments

The NPI-Q is an informant-based assessment tool that measures the presence and severity of behavioural disturbances in 12 behavioural domains of agitation, anxiety, apathy, appetite changes, delusions, depression, disinhibition, abnormally elevated mood, hallucinations, irritability, repetitive motor behaviours, and sleep behaviour changes in clinical settings, within the past month [24]. Higher NPI-Q scores represent greater severity of NPS.

### Estimated years to symptom onset (EYO) of DIAN

The estimated age of onset of cognitive impairment in the cognitively normal individuals from the DIAN was calculated based on the mean mutation age of symptom onset and/or the parental age of symptom onset according to the following steps as described previously [25]:

(i) At any study visit, the EYO was calculated as the age at visit subtracting the mean mutation age of symptom onset if the individual’s mutation is known, which is available in the DIAN database. (ii) If the individual’s mutation is not available in the DIAN database (e.g. the mutation has not been previously reported or other member age of onset not available), then at any study visit, the EYO was calculated as the age at visit subtracting the parental age of symptom onset. The shorter the EYO, the closer the proximity of the individual’s time of clinical disease.

### Genetic analysis

Sequencing of the *APP*, *PSEN1* and *PSEN2* genes was performed by the DIAN Genetics Core investigators as previously described [18], to reveal the presence of disease-causing mutation in individuals at the risk of AD.

### CSF analysis

CSF levels of Aβ_1–42_, total tau (t-tau) and p-tau181 were measured by immunoassay by the DIAN Biomarker Core at the Washington University, using the Luminex bead-based multiplexed xMAP technology (INNO-BIA AlzBio3™, Innogenetics, Ghent, Belgium) as previously described [17].

### MRI and PET methods

MRI and PET standard acquisition protocols have been described in the DIAN website. T1-weighted MRI images corrected for field distortions were processed with the CIVET image processing pipeline [26] and the PET images were processed with an established image processing pipeline described previously [27]. The pre-processed images from the DIAN database were spatially normalized to the Montreal Neurological Institute (MNI) 152 standardized space by using the transformations obtained for PET native to MRI native space and the MRI native to the MNI 152 space. The [^18^F]FDG PET standardized uptake value ratio (SUVR) maps were then generated using the pons as the reference region [28, 29]. The global brain glucose uptake was calculated by averaging the [^18^F]FDG SUVR within several brain regions characteristic to the AD process, including the precuneus, pre-frontal, orbitofrontal, parietal, temporal, anterior, and posterior cingulate cortices.

### Statistical analysis

The descriptive statistics and frequency distributions of baseline demographics, mutation characteristics and CSF AD biomarkers were summarized and compared between DIAD mutation carriers and non-carriers using family-level random-effect models for both continuous and categorical measurements using the STATA 15.0 software. Principal components were derived for the variables NPI-Q and EYO to resolve collinear relationships.

The linear mixed effect models with family-level random effects evaluated the interactions between NPI-Q (total and sub-scale scores individually and as a whole), age and EYO on FDG SUVR in the mutation carriers and non-carriers. We modelled FDG SUVR as a function of the interactions of NPI-Q, age and EYO and covariates, where FDG SUVR_*ij*_ denotes the FDG uptake for the *j*th person from the *i*th family, NPI-Q_*ij*_ indicates the severity of NPS, age_*ij*_ indicates the age of participant at the time of study visit, EYO_*ij*_ indicates the years to estimated age of symptom onset and X_*ij*_ represents fixed effect covariates for gender, education, *APOE* ε4 status and family mutation type (*APP, PSEN1* and *PSEN2*):

$$ \mathrm{FDG}\ {\mathrm{SUVR}}_{ij}\sim {\beta}_0+{\beta}_1\left(\mathrm{NPI}-{\mathrm{Q}}_{ij}\right)+{\beta}_2\left({\mathrm{EYO}}_{ij}\right)+{\beta}_3\left({\mathrm{Age}}_{ij}\right)+{\beta}_4\left(\mathrm{NPI}-{\mathrm{Q}}_{ij}\times {\mathrm{EYO}}_{ij}\right)+{\beta}_5\left(\mathrm{NPI}-{\mathrm{Q}}_{ij}\times {\mathrm{Age}}_{ij}\right)+{\beta}_6\left({\mathrm{EYO}}_{ij}\times {\mathrm{Age}}_{ij}\right)+{\beta}_7\left(\mathrm{NPI}-{\mathrm{Q}}_{ij}\times {\mathrm{EYO}}_{ij}\times {\mathrm{Age}}_{ij}\right)+{\beta}_8\left({\mathrm{X}}_{ij}\right)+{F}_i+{\upvarepsilon}_{ij} $$

where *F*_*i*_ represents a random effect for all individuals from family *i*, and ε_*ij*_ is the residual error assumed to be independent and normally distributed for all individuals.

The family-level random effect accounts for the correlations between individuals within the same family. Although correlations between family members might vary with the relationship type, due to the fairly small sizes of the families, this was modelled with a single random effect.

Voxel-based statistical analyses were then performed using the R Statistical Software Package version 3.3.0 with the RMINC library [30], to test the interactions of NPI-Q, age and EYO on FDG SUVR in the DIAD mutation carriers and non-carriers. All voxel-based regression analyses were corrected for multiple comparisons using random field theory [31] at *p* <  0.001.

Exploratory factor analysis was performed on the sub-components of NPI-Q to identify the neuropsychiatric subsyndromes within the DIAD mutation carriers. This exploratory analysis aims to determine the multidimensional relationships of the NPI-Q sub-components and their overall effects over the individual contributions on the outcomes of interest. Linear mixed effect models with family-level random effects were then used to evaluate the interactions of specific neuropsychiatric subsyndromes and EYO on FDG SUVR in the mutation-carrier group.

## Results

### Baseline demographics, mutation characteristics and CSF AD biomarkers

Two hundred and twenty-one cognitively intact individuals (119 [53.85%] DIAD mutation carriers and 102 [46.15%] non-carriers) were included in this study. Twenty-nine individuals had year 1, 24 had year 2, 46 had year 3, 7 had year 4, 4 had year 5 and 3 had year 6 follow-up data. The baseline demographics, *APOE* ε4 carrier status, and AD CSF biomarker characteristics are summarized in Table 1. The NPI-Q scores among the DIAD mutation carriers and non-carriers are summarized in Table 2. The DIAD mutation carriers and non-carriers did not differ significantly in age, gender, education and *APOE* ε4 status. As expected, the DIAD mutation carriers had lower CSF Aβ_1–42_, as well as higher CSF p-tau181 and CSF t-tau levels than the non-carriers. The NPI-Q total and sub-scale scores did not differ significantly between the mutation carriers and non-carriers.

**Table 1:** Baseline demographics and sample characteristics of cognitively intact DIAD mutation carriers and non-carriers

	DIAD mutation non-carriers (*n* = 102)	DIAD mutation carriers (*n* = 119)	*p* value
Age, years, mean (SD)	38.99 (10.75)	36.45 (9.24)	0.060
Male, *n* (%)	44 (43.13)	58 (48.73)	0.405
Education, years, mean (SD)	15.15 (2.88)	14.92 (3.05)	0.566
*APOE* carrier status			0.600
*APOE* ε2/ε2, ε2/ε3, ε3/ε3 carriers, *n* (%)	72 (70.06)	83 (69.7)	
*APOE* ε2/ε4 carriers, *n* (%)	6 (5.88)	4 (3.36)	
*APOE* ε3/ε4, ε4/ε4 carriers, *n* (%)	24 (23.52)	32 (26.89)	
Parental age of onset, years, mean (SD)	46.85 (6.37)	48.32 (7.25)	0.179
EYO, years, mean (SD)	−9.40 (11.92)	−11.51 (9.40)	0.078
DIAD mutation type			0.863
*APP*, *n* (%)	20 (19.60)	20 (16.80)	
*PS1*, *n* (%)	69 (67.65)	83 (69.75)	
*PS2*, *n* (%)	13 (12.75)	16 (13.45)	
MMSE, mean, (SD)	29.39 (0.83)	29.08 (1.19)	0.052
CSF Aβ_1–42_, mean, pg/ml (SD)^^^	461.14 (138.27)	363.66 (166.72)	< 0.001
CSF p-tau181, mean, pg/ml (SD)^†^	29.64 (11.96)	53.44 (30.65)	< 0.001
CSF t-tau, mean, pg/ml (SD)^†^	54.86 (25.47)	92.06 (62.16)	< 0.001

*P* values were assessed using family-level random-effects models for the continuous variables and categorical variables, taking into account the analysis of multiple family members within the families. *CSF* cerebrospinal fluid, *EYO* estimated years to symptom onset, *MMSE* mini-mental state examination

^^^ CSF Aβ_1–42_ data for the baseline visit were not available for 19 mutation carriers and 23 non-carriers

^†^ CSF p-tau181 & CSF t-tau data for the baseline visit were not available for 18 mutation carriers and 21 non-carriers

**Table 2:** Neuropsychiatric symptoms among cognitively intact DIAD mutation carriers and non-carriers

	DIAD mutation non-carriers (*n* = 102)	DIAD mutation carriers (*n* = 119)	*p* value
NPI-Q Score	0.64 (1.23)	0.88 (2.19)	0.338
NPI-Q Sub-scale			
Agitation	0.07 (0.29)	0.08 (0.40)	0.699
Anxiety	0.10 (0.35)	0.11 (0.33)	0.856
Apathy	0.01 (0.09)	0.04 (0.27)	0.295
Appetite	0.10 (0.41)	0.10 (0.39)	0.525
Delusion	0.00 (0.00)	0.01 (0.09)	–
Depression	0.13 (0.43)	0.13 (0.46)	0.748
Disinhibition	0.01 (0.09)	0.03 (0.27)	0.609
Elation	0.02 (0.13)	0.00 (0.00)	0.055
Hallucination	0.00 (0.00)	0.00 (0.00)	–
Irritability	0.12 (0.40)	0.18 (0.56)	0.207
Motor	0.02 (0.19)	0.05 (0.28)	0.366
Sleep changes	0.07 (0.35)	0.14 (0.47)	0.195

Mixed Model with family-level random-effects, corrected for age, gender, *APOE*, education and family mutation type

*NPI-Q* neuropsychiatric inventory-questionnaire

### Interactions among NPI-Q, EYO and age on global [^18^F]FDG uptake

The interaction between NPI-Q and EYO was significantly associated with global [^18^F]FDG SUVR only in the DIAD mutation carriers. We found that higher NPI-Q and shorter EYO were associated with a more rapid decline of global [^18^F]FDG uptake (β = − 0.029, 95% CI − 0.054 to − 0.004, *p* = 0.024) in the DIAD mutation carriers but not in the non-carriers (β = − 0.008, 95% CI − 0.044 to 0.027, *p* = 0.641) (Table 3). The NPI-Q sub-scales whose interaction with EYO was significantly associated with global [^18^F]FDG uptake were anxiety, apathy, depression and irritability for the mutation group. Upon including all the sub-scales in one multivariate model, no significant interactions were observed. We did not find a statistically significant association of interaction between NPI-Q and age with global [^18^F]FDG uptake in either DIAD mutation carriers or non-carriers. We also did not find a statistically significant association of interaction between NPI-Q, age and EYO with global [^18^F]FDG uptake in either DIAD mutation carriers or non-carriers.

**Table 3:** Associations of interactions of NPI-Q and EYO with global [^18^F]FDG uptake in cognitively intact DIAD mutation carriers and non-carriers

	DIAD mutation non-carriers			DIAD mutation carriers		
	Regression estimate	95% CI	*p* value	Regression estimate	95% CI	*p* value
NPI-Q	− 0.013	− 0.040, 0.014	0.345	− 0.012	− 0.030, 0.006	0.195
Age	− 0.054	− 0.085, − 0.022	0.001	− 0.078	− 0.108, − 0.048	< 0.001
EYO	− 0.020	− 0.050, 0.010	0.186	− 0.073	− 0.105, − 0.041	< 0.001
NPI-Q X EYO	− 0.008	− 0.044, 0.027	0.641	− 0.029	− 0.054, − 0.004	0.024
NPI-Q subscales						
Agitation	0.004	− 0.017, 0.025	0.406	0.004	− 0.018, 0.027	0.690
Age	− 0.057	− 0.088, − 0.026	< 0.001	− 0.077	− 0.107, − 0.046	< 0.001
EYO	− 0.020	− 0.050, 0.009	0.170	− 0.076	− 0.110, − 0.043	< 0.001
Agitation X EYO	− 0.012	− 0.028, 0.005	0.164	− 0.004	− 0.020, 0.011	0.600
Anxiety	− 0.009	− 0.036, 0.017	0.498	− 0.011	− 0.028, 0.006	0.202
Age	− 0.055	− 0.086, − 0.024	0.001	− 0.074	− 0.104, − 0.043	< 0.001
EYO	− 0.016	− 0.047, 0.014	0.282	− 0.077	− 0.110, − 0.043	< 0.001
Anxiety X EYO	− 0.013	− 0.039, 0.013	0.310	− 0.029	− 0.054, − 0.005	0.020
Apathy	0.254	− 0.028, 0.535	0.076	− 0.107	− 0.026, 0.005	0.169
Age	− 0.018	− 0.066, 0.030	0.465	− 0.079	− 0.110, − 0.049	< 0.001
EYO	− 0.059	− 0.111, − 0.008	0.024	− 0.072	− 0.104, − 0.039	< 0.001
Apathy X EYO	− 0.308	− 0.631, 0.015	0.061	− 0.027	− 0.044, − 0.010	0.002
Appetite	− 0.006	− 0.025, 0.012	0.497	− 0.005	− 0.024, 0.013	0.575
Age	− 0.056	− 0.087, − 0.025	0.001	− 0.076	− 0.107, − 0.045	< 0.001
EYO	− 0.020	− 0.050, 0.010	0.181	− 0.076	− 0.109, − 0.053	< 0.001
Appetite X EYO	0.014	− 0.005, 0.033	0.142	0.005	− 0.011, 0.021	0.526
Delusion	− 0.136	− 0.325, 0.053	0.154	− 0.080	− 0.422, 0.263	0.646
Age	− 0.056	− 0.086, − 0.025	0.001	− 0.082	− 0.112, − 0.051	< 0.001
EYO	− 0.017	− 0.046, 0.012	0.254	− 0.067	− 0.099, − 0.034	< 0.001
Delusion X EYO	− 0.022	− 0.051, 0.008	0.147	0.003	− 0.029, 0.035	0.844
Depression	0.014	− 0.011, 0.040	0.281	− 0.213	− 0.038, − 0.004	0.015
Age	− 0.053	− 0.084, − 0.022	0.001	− 0.080	− 0.110, − 0.050	< 0.001
EYO	− 0.026	− 0.057, 0.005	0.093	− 0.078	− 0.090, − 0.045	0.001
Depression X EYO	0.012	− 0.033, 0.009	0.270	− 0.042	− 0.064, − 0.019	< 0.001
Disinhibition	0.237	− 0.007, 0.480	0.056	0.345	− 0.016, 0.085	0.180
Age	− 0.032	− 0.069, 0.003	0.092	− 0.080	− 0.111, − 0.050	< 0.001
EYO	− 0.049	− 0.090, − 0.007	0.022	− 0.087	− 0.126, − 0.048	< 0.001
Disinhibition X EYO	− 0.324	− 0.642, 0.006	0.078	− 0.148	− 0.372, 0.007	0.196
Irritability	0.001	− 0.022, 0.024	0.948	0.004	− 0.013, 0.021	0.619
Age	− 0.048	− 0.080, − 0.017	0.003	− 0.081	− 0.111, − 0.051	< 0.001
EYO	− 0.023	− 0.022, 0.007	0.133	− 0.077	− 0.016, − 0.044	< 0.001
Irritability X EYO	− 0.023	− 0.045. 0.001	0.078	− 0.028	− 0.048, − 0.008	0.006
Motor	− 0.518	− 1.47, 0.535	0.332	− 0.138	− 0.313, 0.004	0.123
Age	− 0.579	− 2.37, 1.22	0.324	− 0.078	− 0.109, − 0.047	< 0.001
EYO	− 0.109	− 0.190, − 0.011	0.028	− 0.071	− 0.104, − 0.037	< 0.001
Motor X EYO	− 0.370	− 0.818, 0.079	0.106	− 0.018	− 0.048, 0.013	0.263
Sleep changes	− 0.001	− 0.032, 0.029	0.928	− 0.008	− 0.024, 0.007	0.279
Age	− 0.054	− 0.086, − 0.023	0.001	− 0.075	− 0.106, − 0.045	< 0.001
EYO	− 0.019	− 0.049, 0.011	0.203	− 0.074	− 0.107, − 0.041	< 0.001
Sleep changes X EYO	− 0.015	− 0.091, 0.017	0.317	− 0.009	− 0.011, 0.013	0.424

Mixed Model with family-level random-effects, corrected for 2-way interactions (NPI-Q sub-scales × Age & Age × EYO), 3-way interaction (NPI-Q sub-scales × Age × EYO), gender, *APOE*, education and family mutation type

### Associations of NPI-Q and EYO with regional [^18^F]FDG uptake

**Fig. 1:**
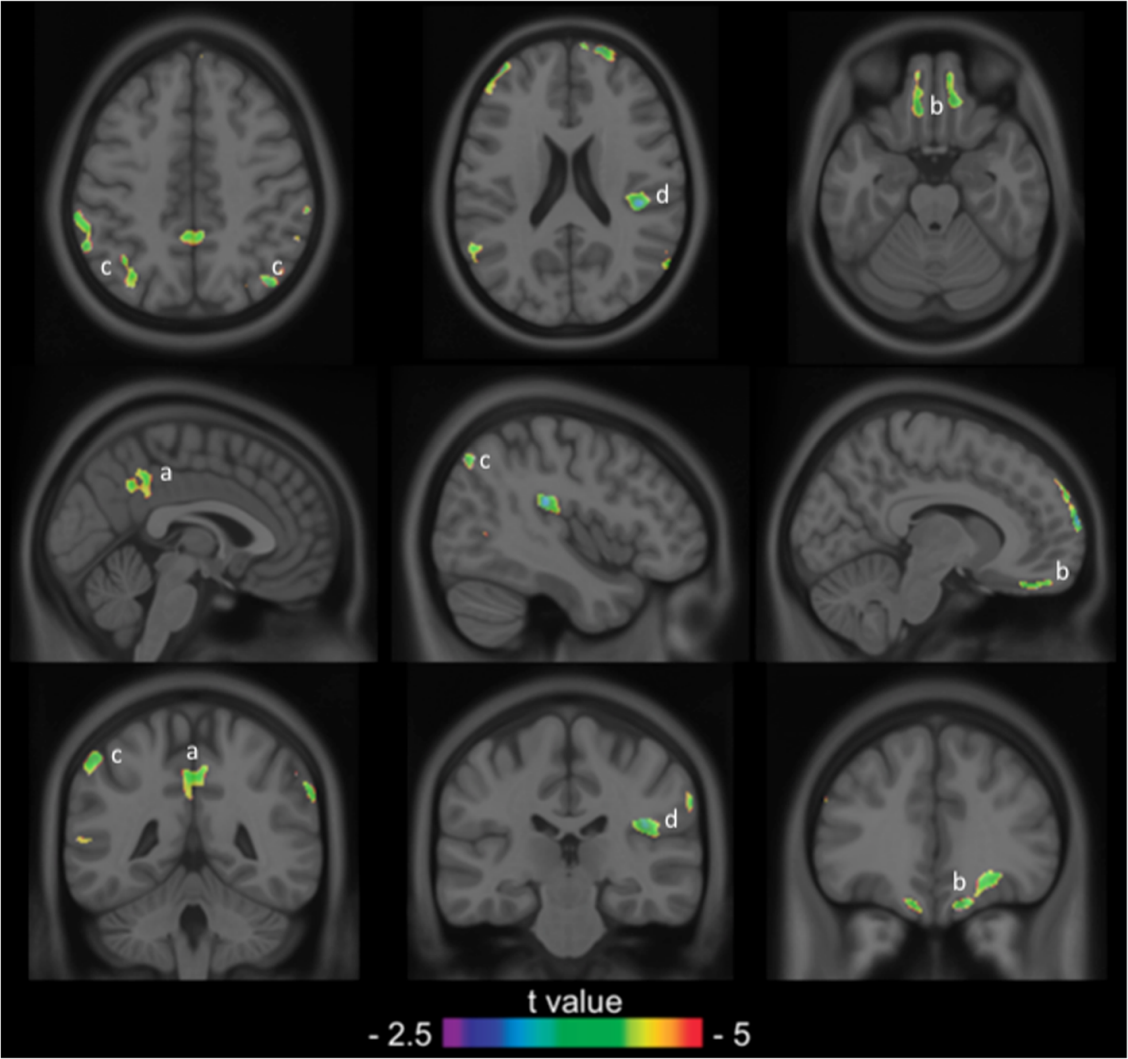
Higher NPI-Q and shorter EYO of AD were associated with higher [^18^F]FDG uptake decline in DIAD mutation carriers. Statistical parametric map overlaid on a structural MRI scan shows regions in the PCC (**a**), vmPFC (**b**), bilateral parietal lobes (**c**) and right AI (**d**) where higher [^18^F]FDG uptake decline was found in cognitively normal DIAD mutation carriers with higher NPI-Q scores and shorter EYO of AD. The analysis was corrected for gender, education, *APOE* ε4 status and family mutation type (*APP, PSEN1* and *PSEN2*) and multiple comparisons were corrected using random field theory at *p* <  0.001. AD: Alzheimer disease; AI: anterior insula; DIAD: dominantly inherited Alzheimer’s disease; EYO: estimated years to onset; [^18^F]FDG: [^18^F]fluorodeoxyglucose; NPI-Q: Neuropsychiatric Inventory; PCC: posterior cingulate cortex; vmPFC: ventromedial prefrontal cortex

The voxel-based analysis indicated that the interaction between higher NPI-Q and shorter EYO was associated with regional [^18^F]FDG uptake decline in the posterior cingulate cortex (PCC), ventromedial prefrontal cortex (vmPFC), bilateral parietal lobes and right insula in DIAD mutation carriers (Fig. 1). There was no statistically significant association of interaction between NPI-Q and EYO, with regional [^18^F]FDG uptake in DIAD mutation non-carriers. There was no statistically significant association of interactions between NPI-Q and age, and among NPI-Q, age and EYO, with regional [^18^F]FDG uptake, in either DIAD mutation carriers or non-carriers.

### Factor analysis of NPI-Q subcomponents

A four-factor solution was applied to the NPI-Q sub-components, which explained 70% of the variance. The neuropsychiatric subsyndromes derived from the factor analysis were (1) agitation, disinhibition, irritability and depression; (2) anxiety, apathy, depression, motor behaviours, and sleep behaviour changes; (3) delusion, sleep behaviour changes and irritability; and (4) appetite and anxiety (Table 4). After including all the subsyndromes in one multivariate model (with family-level as random-effects, adjusted for gender, *APOE*, education and family mutation type), only the interaction of the neuropsychiatric subsyndromes agitation, disinhibition, irritability and depression with shorter EYO was associated with the decline of global [^18^F]FDG uptake (β = − 0.044, 95% CI − 0.071 to − 0.017, *p* = 0.001) in the DIAD mutation carriers (Table 5).

**Table 4:** Factors of neuropsychiatric subsyndromes of the cognitively intact DIAD mutation carriers

	Neuropsychiatric Subsyndromes			
	1	2	3	4
Agitation	0.867			
Anxiety		0.477		0.421
Apathy		0.785		
Appetite				0.929
Delusion			0.908	
Depression	0.456	0.577		
Disinhibition	0.822			
Irritability	0.692		0.410	
Motor		0.797		
Sleep Changes		0.489	0.564	

Factor analysis identified the above 4 components which explained 70% of the variance

**Table 5:** Neuropsychiatric symptoms (Factors) among cognitively intact DIAD mutation carriers

	Regression estimate	95% CI	*p* value
Age	− 0.076	− 0.016, − 0.002	< 0.001
EYO	− 0.070	− 0.101, − 0.038	< 0.001
Factor 1	Agitation + Disinhibition + Irritability + Depression		
	0.010	− 0.024, 0.022	0.367
Factor 2	Anxiety + Apathy + Depression + Motor + Sleep changes		
	− 0.026	− 0.049, − 0.004	0.023
Factor 3	Delusion + Sleep changes + Irritability		
	0.008	− 0.014, 0.031	0.478
Factor 4	Appetite + Anxiety		
	− 0.004	− 0.020, 0.012	0.481
2-Way Interactions			
Factor 1 X EYO	− 0.044	− 0.071, 0.017	0.001
Factor 2 X EYO	− 0.020	− 0.047, 0.007	0.140
Factor 3 X EYO	0.006	− 0.020, 0.033	0.637
Factor 4 X EYO	− 0.005	− 0.026, 0.016	0.649

Mixed Model with family-level random-effects, corrected for 2-way interactions of Factors (1 to 4) X age, 3-way interactions Factors (1 to 4) X Age X EYO, gender, *APOE*, education and family mutation type

## Discussion

The present study showed that NPS may be early clinical manifestations of subsequent metabolic dysfunction in brain regions susceptible to AD pathophysiology in cognitively intact DIAD mutation carriers. In these individuals who were destined to develop AD, the more severe the NPS and the shorter the EYO to AD dementia onset, the more rapid the metabolic decline in the PCC, vmPFC, bilateral parietal lobes and right insular. We found that the interaction of the neuropsychiatric subsyndromes agitation, disinhibition, irritability and depression with shorter EYO was associated with a decline of global metabolic uptake over time.

Accumulating evidence has demonstrated the importance of NPS as predictors of cognitive decline in cognitively normal individuals. In the population-based Mayo Clinic Study of Aging, the presence of NPS such as agitation, apathy, anxiety, irritability and depression at baseline increased the risk of incident MCI compared to those without NPS [8]. In the Alzheimer’s Disease Cooperative Study Prevention Instrument Project, anxiety and depression at baseline predicted CDR conversion to ≥0.5 in cognitively intact older subjects over a 4-year follow-up [32], while among the National Alzheimer’s Coordinating Centers cognitively normal (NACC) volunteers, over 59% developed NPS before the diagnosis of any cognitive disorder, with depression and irritability being the most common NPS to precede cognitive diagnoses [10]. NPS among cognitively normal individuals may also represent an early manifestation of progressive metabolic dysfunction. In cognitively normal persons aged  >  70 years, depressive and anxiety symptoms are associated with decreased FDG uptake in AD-related regions [33]. In a recent study of preclinical sporadic AD individuals, we found that NPS driven by sleep behavior and irritability domains were associated with metabolic dysfunctions in the limbic network and predicted hypometabolism in the PCC [13]. Therefore, our present findings further support the emerging conceptual framework that NPS are early non-cognitive manifestations of subsequent metabolic decline in AD.

The default mode network (DMN), which comprises the PCC, vmPFC and inferior parietal lobes, plays a vital role in episodic memory processing and decreased metabolism in the DMN is observed early in AD [34–36]. The salience network (SN), which is critical in detecting and integrating behavioural and emotional stimuli, has key nodes in the insular cortex and modulates the switch between the DMN and the central executive network [37, 38]. Impairment of the SN can lead to numerous neuropsychiatric disorders such as psychosis [39] and depression [40]. Brain metabolic dysfunctions within the SN are also related to NPS in AD [41]. Therefore, our findings of the association between NPS and more rapid FDG uptake decline in the PCC, vmPFC, parietal lobes, and right insula in DIAD mutation carriers with shorter EYO of AD dementia support the link between early NPS and limbic structures and brain regions involved in early AD pathophysiology.

While there is heterogeneity in the neuropsychiatric manifestations in AD, certain NPS tend to co-express. Hence, several neuropsychiatric subsyndromes have been identified to characterise the clustering of NPS [42] in AD. In our study, the exploratory factor analysis revealed four neuropsychiatric subsyndromes (Table 4) and among them, only the neuropsychiatric subsyndrome “agitation, disinhibition, irritability and depression” was associated with metabolic decline in cognitively intact DIAD mutation carriers with shorter EYO of AD dementia. This is consistent with findings from a systemic review of behavioural and psychological subsyndromes among elderly individuals with sporadic AD, where 34 different clusters were found and agitation/aggression, depression, anxiety and irritability were most commonly clustered together [43]. In addition, delusion and hallucinations, depression and anxiety, agitation and irritability, and euphoria and disinhibition tend to be frequently associated symptoms. Hence, our finding is consistent with the current evidence of neuropsychiatric subsyndromes in AD. In addition, given that the currently reported subsyndromes are mostly defined among elderly individuals with sporadic AD, our results further advanced the field by identifying specific neuropsychiatric subsyndromes among younger cognitively intact DIAD mutation carriers who are destined to develop AD. However, this will need to be confirmed in future studies in a different cohort using different methodological approaches.

The neurobiology of agitation, disinhibition, irritability and depression is closely linked to dysfunctions within the PCC, vmPFC, bilateral parietal lobes and right insular. The vmPFC regulates behavioural responses such as changing reinforcement contingencies and emotional processing and regulation [44, 45]. Irritability is linked to abnormal emotional processing associated with vmPFC and PCC, while behavioral disinhibition and prominent emotional lability are linked to lesions in the vmPFC. Dysfunctions within the vmPFC and PCC, which are part of the neural network involved in the modulation of normal emotional behaviour, also lead to affective disorders and depressive symptoms [46]. The insular plays a key role in producing appropriate behavioral responses in a person by integrating affective, homeostatic, and higher-order cognitive processes [47]. Irritability is associated with dysfunctions within the insular [48] while AD patients with agitation also appear to have dysfunctions within the frontal cortex, anterior cingulate cortex, orbitofrontal cortex, amygdala, and insula [49]. While we found that the neuropsychiatric subsyndrome of agitation, disinhibition, irritability and depression is linked to regional metabolic decline in cognitively normal DIAD mutation carriers, further studies are needed to evaluate if this subsyndrome also identifies cognitively normal individuals with an increased risk of pathological progression in sporadic AD.

The main strength of the present longitudinal study was the inclusion of preclinical DIAD mutation carriers who had AD pathology and were destined to develop AD in future. This allows the study of associations between NPS, metabolism and effects of increasing AD pathology over time (EYO). Furthermore, given that individuals may be susceptible to NPS presentations due to genetic, family and environmental factors, or being at risk for DIAD, employing both DIAD mutation carriers and non-carriers enables the control of these factors.

There were limitations in our study. First, while NPI-Q is commonly used to detect NPS in AD patients, the NPI-Q is not developed for patients with prodromal or preclinical AD. Certain items of the NPI-Q may also be more relevant than others in a young cognitively intact cohort. Hence, the sensitivity of NPI-Q in identifying early NPS in cognitively intact individuals remains unclear. In addition, given that the NPI-Q is based on responses from an informed caregiver, the NPI-Q scores may not accurately reflect the NPS of study participants. In this regard, the mild behavioral impairment checklist (MBI-C) [50, 51] is a 34-item instrument that is sensitive in detecting MBI in people with MCI, a construct that characterises the emergence of sustained NPS in pre-dementia populations as a precursor to cognitive decline and dementia. Other scales that are potentially relevant include the Hospital Anxiety and Depression Scale (HADS) [52] and the Depression Anxiety Stress-Scale (DASS) [53]. Future studies of NPS in pre-dementia individuals should consider including these scales. Second, the NPI-Q total and sub-scale scores of our study population were relatively low, which may be due to the limitation of the NPI-Q in measuring NPS in cognitively intact individuals. While our findings may highlight the relevance of NPS with metabolism, where a small severity of NPS is associated with a big impact on metabolic decline, this needs to be confirmed in future studies. Third, while examining the associations of NPS with metabolic decline in a cohort of DIAD mutation carriers who were destined to develop AD, we did not specifically study the associations between NPS and AD biomarkers such as beta-amyloid and tau. Given the emerging evidence showing that NPS are associated with AD pathophysiology in preclinical and MCI individuals [4, 54], future longitudinal studies are needed to determine this relationship. Last, there may be potential hazards of interpreting statistical constructs as theoretical constructs in the factor analytic literature [55]. Furthermore, a systemic review has found a relatively low concordance of the composition of NPI clusters among available evidence although some consistent associations of specific symptoms defining potential subsyndromes in AD across studies had been observed [56]. Acknowledging this limitation, a novel version of principal component analysis that mitigates excessive floor effects in NPI scores has been developed for more robust identification of neurobehavioral subsyndromes [57]. Therefore, future studies could use this approach to test the replicability of the associations between neurobehavioral subsyndromes and metabolic decline reported in this study.

## Conclusion

Our findings support the emerging conceptual framework that NPS may be early clinical presentations of AD pathophysiology progression. Given that early NPS may contribute to the characterization of the preclinical AD stage, cognitively intact individuals presenting with NPS can be identified earlier so as to allow a personalized and timely preventive intervention.

## Data Availability

The datasets used and/or analysed during the current study are available from the Dominantly Inherited Alzheimer Network on reasonable request.

## Abbreviations

AD: Alzheimer’s disease
CDR: Clinical dementia rating
CSF: Cerebrospinal fluid
DIAD: Dominantly inherited AD
DIAN: Dominantly Inherited Alzheimer’s Network
EYO: Estimated years to symptom onset
FDG: [^18^F]Flurodeoxyglucose
MBI: Mild behavioral impairment
MBI-C: Mild behavioral impairment checklist
MCI: Mild cognitive impairment
NACC: National Alzheimer’s Coordinating Center
NPI-Q: Neuropsychiatric inventory-questionnaire
NPS: Neuropsychiatric symptoms
PCC: Posterior cingulate cortex
PET: Positron emission tomography
SUVR: Standardized uptake value ratio
vmPFC: Ventral medial prefrontal cortex
